# Prevalence and impact of long COVID-19 among patients with diabetes and cardiovascular diseases in Bangladesh

**DOI:** 10.3389/fpubh.2023.1222868

**Published:** 2023-10-27

**Authors:** Nadim Sharif, Nazmul Sharif, Afsana Khan, Ibrahim F. Halawani, Fuad M. Alzahrani, Khalid J. Alzahrani, Isabel De la Torre Díez, Debora Libertad Ramírez Vargas, Angel Gabriel Kuc Castilla, Anowar Khasru Parvez, Shuvra Kanti Dey

**Affiliations:** ^1^Department of Microbiology, Jahangirnagar University, Savar, Bangladesh; ^2^Department of Mathematics, Rajshahi University of Engineering & Technology, Rajshahi, Bangladesh; ^3^Department of Statistics, Jahangirnagar University, Savar, Bangladesh; ^4^Department of Clinical Laboratories Sciences, College of Applied Medical Sciences, Taif University, Taif, Saudi Arabia; ^5^University of Valladolid, Valladolid, Spain; ^6^Universidad Internacional Iberoamericana, Campeche, Mexico; ^7^Universidade Internacional do Cuanza, Kuito, Angola; ^8^Fundación Universitaria Internacional de Colombia, Bogotá, Colombia; ^9^Universidad Europea del Atlántico, Santander, Spain; ^10^Universidad Internacional Iberoamericana, Arecibo, PR, United States

**Keywords:** long-COVID-19, comorbidity, diabetes, cardiovascular disease, Bangladesh

## Abstract

**Introduction:**

Co-prevalence of long-COVID-19, cardiovascular diseases and diabetes is one of the major health challenges of the pandemic worldwide. Studies on long-COVID-19 and associated health outcomes are absent in Bangladesh. The main aim of this study was to determine the prevalence and impact of long-COVID-19 on preexisting diabetes and cardiovascular diseases (CVD) on health outcomes among patients in Bangladesh.

**Methods:**

We collected data from 3,250 participants in Bangladesh, retrospectively. Multivariable logistic regression model was used to determine the odds ratio between independent and dependent variables. Kaplan-Meier survival curve was used to determine the cumulative survival.

**Results:**

COVID-19 was detected among 73.4% (2,385 of 3,250) participants. Acute long-COVID-19 was detected among 28.4% (678 of 2,385) and chronic long-COVID-19 among 71.6% (1,707 of 2,385) patients. CVD and diabetes were found among 32%, and 24% patients, respectively. Mortality rate was 18% (585 of 3,250) among the participants. Co-prevalence of CVD, diabetes and COVID-19 was involved in majority of fatality (95%). Fever (97%), dry cough (87%) and loss of taste and smell (85%) were the most prevalent symptoms. Patients with co-prevalence of CVD, diabetes and COVID-19 had higher risk of fatality (OR: 3.65, 95% CI, 2.79–4.24). Co-prevalence of CVD, diabetes and chronic long-COVID-19 were detected among 11.9% patients.

**Discussion:**

Risk of hospitalization and fatality reduced significantly among the vaccinated. This is one of the early studies on long-COVID-19 in Bangladesh.

## 1. Introduction

In Bangladesh, COVID-19 have become one of the major public health concerns since the first report on March, 2020. COVID-19 has spread faster and affected people of all aspects in Bangladesh (1, 2). Nearly 2 038 129 people have been infected with COVID-19 and 29 446 deaths have been reported till April 24, 2023 in Bangladesh (3, 4). About 88.5% of the total population of Bangladesh has received at least one dose and 48% got 3rd dose of vaccine (1, 3). Three peaks of COVID-19 pandemic have been identified in Bangladesh since 2020. One peak was apparently confined within the period of March, 2021 to May, 2021, and another one during June, 2021 to September, 2021 and the last one during January, 2022 to March, 2022 (3, 4). Though vaccination is ongoing, breakthrough cases and variants with escape mutations are continuously contributing to the increase of cases of COVID-19 in Bangladesh. As of daily update on 24th April, 2023 the number of active cases were 10 197 and 254 of them had critical health conditions (3–5).

Clinical features of post-COVID-19 and health conditions among the patients are highly heterogenous, complex and not well-characterized (6). According to Fernández-de-las-Peñas et al. long post-COVID-19 is defined as cases having symptoms from week 12 to week 24 after (6, 7). According to WHO, “post COVID-19 or long COVID condition refers to long-term symptoms after having COVID-19”. However, the current knowledge suggests drawback of this definition. Based on the fluctuation of post-COVID-19 symptoms on patients, long COVID-19 should be monitor for newer and persistent symptoms over weeks, months and years. According to the most accepted definition of long COVID-19, post-acute sequelae is defined as any symptoms of COVID-19 after recovery of infection and lasting for more than 5 to 12 weeks and chronic post-COVID-19 symptoms lasting for more than 12 weeks (6, 7). A significant proportion of COVID-19 patients have been suffering from acute and chronic long-COVID-19 (6–8). Existing data suggests that presence of pre-existing diseases including diabetes, hypertension, cardiovascular diseases (CVD), autoimmune diseases, obesity, and chronic obstructive pulmonary disease (COPD) among COVID-19 patients contribute to serious health outcome, hospitalization and ICU admission (9–11). Further, patients with long COVID-19 and preexisting health conditions have poor prognosis and high fatality rate (8–10). Studies suggest that COVID-19 has the ability to influence the onset of specific type of diabetes among non-diabetic patients (11, 12). Presence of long-COVID-19 have worsened the health condition and disease prognosis of diabetes, CVD and COPD over long time (8–11). Prevalence of diabetes among COVID-19 patients varied between 9 and 45% and CVDs from 7 to 45% in Bangladesh, UK, China, USA, India and Italy (12–19). Studies have suggested that COVID-19 patients with diabetes, COPD and CVDs have higher risk of fatality and developing severe health condition (12–19).

Studies on the prevalence of acute and chronic long-COVID-19 and their relation with pre-existing health conditions on the outcome are lacking in Bangladesh. Early studies on the impact of COVID-19 and comorbidities on the health outcome among patients in Bangladesh also suggest significant relationship of these health conditions. Therefore, we conducted this study to investigate the impact of long-COVID-19 among patients with diabetes and CVDs on health outcomes in Bangladesh.

## 2. Materials and methods

### 2.1. Study design and population

A retrospective study was designed. Data was collected from 3,250 participants from seven divisions in Bangladesh during 01 January, 2022 and 31 December, 2022. The age of the participants ranged from 20 years to 78 years. According to the guidelines of the World Health Organization, the diagnosis was conducted by RT-PCR method (20). Data were collected in four sampling frames. Data on the report of hospitalization, ICU admission and discharge were collected directly from the patients and hospital authorities. Death reports were collected from the authorities and confirmed from the relatives of the patients.

### 2.2. Ethical approval

This study was ethically approved by the Biosafety, Biosecurity and Ethical Committee (BBEC) of Jahangirnagar University. Informed consent was taken from patients or relatives of the patients. The protocol number approved by the ethics committee is BBEC, JU/M 2021/COVID-19/(2)1.

### 2.3. Data collection

According to the guidelines of the World Health Organization, nasal or pharyngeal swab specimens were collected and used for the test in the hospitals/clinics (20). A positive outcome was defined by a positive laboratory test in the real-time reverse-transcriptase– PCR (RT-PCR) assay for SARS-CoV-2 and confirmed by high throughput sequencing (20). Data on the sociodemographic and economic conditions including sex, age, origin, monthly income, residing place, occupation, medical history, complication, treatment received (antiviral, antibiotic, steroid therapies, immune therapy, plasma therapy, respiratory support by mechanical ventilation and ICU support) were collected from the patients. Any pre-existing health conditions (defined by the International Classification of Diseases, 10th Revision, Clinical Modification), and outcome were included in this study (16, 19). Data on the pre-existing health conditions including diabetes mellitus, cardiovascular disease (CVD), hypertension, hyperlipidemia, chronic obstructive pulmonary disease (COPD), malignancy, obesity and autoimmune disease were taken from the patients and examined by two experts.

### 2.4. Outcomes

The primary outcome was long time illness associated with COVID-19 infection. Secondary outcomes included hospitalization, admission to ICU, requirement of mechanical ventilation and fatality. Presence of chronic long-COVID-19 have worsened the clinical outcomes of pre-existing CVD, diabetes and COPD. Cardiovascular disease, malignant arrhythmia, diabetes and acute myocardial injury were defined based on the published works (16–19).

### 2.5. Statistical analyses

Percentage, rate and frequency were used for representing the categorical variables. Mean and standard deviation were used for representing central tendency of continuous variables. Independent sample *t*-tests were performed with 95% confidence intervals. *P* < 0.05 was considered statistically significant. The relationship between comorbidities and long COVID-19 were determined. Multivariable logistic-regression analysis was conducted to determine the impacts of sociodemographic factors and comorbidities on health outcome including fatality, hospitalization, ICU and long COVID-19. With 95% confidence intervals, adjusted odds ratios were determined. For the pre-existing comorbidities, Charlson Comorbidity Index (CCI) were computed. We determined the Kaplan-Meier survival estimate by considering different age, sex, and comorbidities among patients with long COVID-19. All of the statistical analyses were performed by International Business Machines (IBM) Statistical Package for the Social Sciences (SPSS) version 28.0 (Chicago, IL, USA) and Microsoft Excel 2021.

## 3. Results

### 3.1. Sociodemographic characteristics of the participants with COVID-19

This study included 3,250 participants from seven divisions in Bangladesh. Nearly 73.4% (2,385 of 3,250) of the participants were COVID-19 positive. The mean (SD) age of the study population was 49 ± 3.6 years. Majority of the participants (66.5%) aged above 40 years (Table 1). The ratio of male to female was 2,340:910 (about 2.6:1). Majority of the participants (65.1%) were from semi-urban and rural areas with poor health facilities. About 96% of the population were from native Bangladeshi. Majority of the participants (68.9%) had a monthly income below 50,000 Bangladeshi taka (500 USD). The availability of health facility and effective treatment varied significantly on monthly income and place of residence in Bangladesh (Table 1).

**Table 1:** Socio-demographic characteristics of study participants.

**Variables**	**Male (%)**	**Female (%)**	**Total (%)**
Study population	2,340/3,250 (74)	910/3,250 (26)	3,250/3,250 (100)
**Age (in years)**			
20–29	290/408 (71)	118/408 (29)	408/3,250 (12)
30–39	510/680 (75)	170/680 (25)	680/3,250 (21)
40–49	460/639 (72)	179/639 (28)	639/3,250 (20)
50–59	382/502 (76)	120/502 (24)	502/3,250 (15)
60–69	358/543 (66)	185/543 (36)	543/3,250 (17)
Above 70	340/478 (71)	138/478 (29)	478/3,250 (15)
**Origin**			
Bangladeshi	2,243/3,125 (72)	882/3,125 (28)	3,125/3,250 (96)
Non-Bangladeshi	97/125 (78)	28/125 (22)	125/3,250 (4)
**Monthly income (Bangladeshi Taka)**			
< 20,000	974/1,284 (76)	310/1,284 (24)	1,284/3,250 (39)
20,000–49,999	639/956 (67)	317/956 (33)	956/3,250 (29)
50,000–100,000	492/665 (74)	173/665 (26)	665/3,250 (21)
>100,000	235/345 (68)	110/345 (32)	345/3,250 (11)
**Employment**			
Employed	1,365/1,780 (77)	415/1,780 (23)	1,780/3,250 (55)
Unemployed	975/1,470 (66)	495/1,470 (34)	1,470/3,250 (45)
**Residence**			
Urban	823/1,128 (73)	305/1,128 (27)	1,128/3,250 (35)
Semi-urban	754/924 (82)	170/924 (18)	924/3,250 (28)
Rural	965/1,198 (81)	233/1,198 (19)	1,198/3,250 (37)

The percentage values represented male and female distributed in different groups.

### 3.2. Prevalence of acute long-COVID-19 and chronic long-COVID-19 in Bangladesh

According to the definition of acute long-COVID-19 and chronic long-COVID-19, we determined the prevalence among the patients. Data were collected from the patients during 2nd week to 24th week after the first appearance of RT-PCR positive test for COVID-19. Acute long-COVID-19 (symptoms within 5–12 weeks) was detected among 28.4% (678 of 2,385) of the COVID-19 positive participants. Acute long-COVID-19 was most prevalent among patients aged 30–39 years (189 of 531) followed by 40–49 years (137 of 528) and 20–29 years (131 of 445), respectively (Table 2). Chronic long-COVID-19 (symptoms after 12 weeks) was found among 71.6% (1,707 of 2,385) patients with COVID-19. Longitudinal analyses showed that after recovery from infection, symptoms of COVID-19 persisted for 13–24 weeks among 29%, 25–48 weeks among 25.6% and >48 weeks among 17% of the patients (Table 2).

**Table 2:** Duration of symptoms after recovery from COVID-19 infection among patients of different age group in Bangladesh.

**Age in years**	**5–12 weeks (%)**	**13–24 weeks (%)**	**25–48 weeks (%)**	**>48 weeks (%)**	**Total (%)**
20–29	131/445 (29.4)	130/445 (29.2)	114/445 (25.6)	70/445 (15.7)	445/2,385 (18.7)
30–39	189/531 (35.6)	141/531 (26.6)	117/531 (22.0)	84/531 (15.8)	531/2,385 (22.3)
40–49	137/528 (25.9)	132/528 (25.0)	137/528 (25.9)	122/528 (23.1)	528/2,385 (22.1)
50–59	95/293 (32.4)	81/293 (27.6)	65/293 (22.2)	52/293 (17.7)	293/2,385 (12.3)
60–69	85/352 (24.1)	111/352 (31.5)	103/352 (29.3)	53/352 (15.1)	352/2,385 (14.8)
Above 70	41/236 (17.4)	97/236 (41.1)	74/236 (31.4)	24/236 (10.2)	236/2,385 (9.9)
Total	678/2,385 (28.4)	692/2,385 (29.0)	610/2,385 (25.6)	405/2,385 (17.0)	
	Acute long-COVID-19 (28.4)	Chronic long-COVID-19 (71.6%)			

The percentage values in bracket represented the persistence of symptoms among the participants distributed in different age group.

### 3.3. Characterization of clinical symptoms and pre-existing health conditions

**FIGURE 1:**
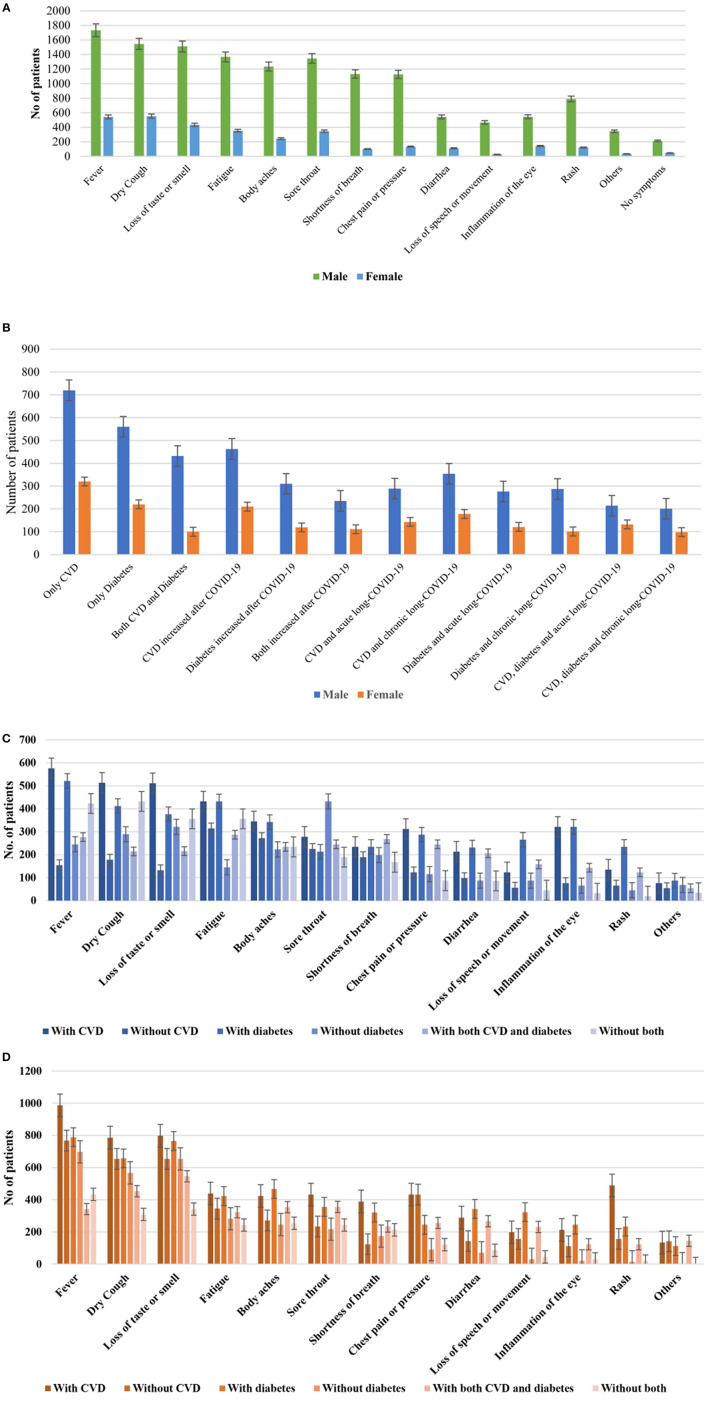
Frequency distribution of **(A)** different clinical symptoms in male and female, **(B)** different comorbidities in male and female patients with COVID-19 in Bangladesh, **(C)** distribution of clinical symptoms among patients with acute long-COVID-19, **(D)** patients with chronic long-COVID-19. Lines in each bar represented confidence intervals.

Clinical symptoms were analyzed by three physicians, recorded separately and compiled. Collected symptoms were cross-checked and evaluated for correction. Fever (97%, 2,313 of 2,385) was the most prevalent symptom followed by dry cough (87%, 2,074 of 2,385), loss of taste or smell (85%, 2,027 of 2,385), fatigue (81%, 1,932 of 2,385), sore throat (79%, 1,884 of 2,385), body aches (72%, 1,717 of 2,385), and chest pain or pressure (56%, 1,335 of 2,385), respectively (Figure 1A). Co-prevalence of multiple symptoms and duration of illness increased with increasing age among the patients. Reappearance of symptoms after 2 weeks of negative of COVID-19 infection were common among the participants (73.4%, 2,385 of 3,250). Male patients had two times greater risk of developing different symptoms associated with COVID-19 than female.

Among the pre-existing health conditions, cardiovascular (CVD) disease was the most prevalent (32%, 1,040 of 3,250) followed by diabetes (24%, 780 of 3,250; Type 2 diabetes mellitus was 63.7% and Type 1 was 36.3%). We detected at least 532 (16.4%) patients of COVID-19 had both CVD and diabetes at the same time (Table 3). Health complications associated with CVD increased among 673 of 1,040 (64.7%) patients and diabetes among 429 of 780 (55%) after patients getting COVID-19 infection. Co-prevalence of CVD, diabetes and acute long-COVID-19 was found among 11% (359 of 3,250) patients. Further, co-prevalence of CVD, diabetes and chronic long-COVID-19 were detected among 11.9% (387 of 3,250) patients (Table 3). Distribution of CVD, diabetes, acute long-COVID-19 and chronic long-COVID-19 were higher among male than female (Figure 1B). Nearly, 7.1% (231 of 2,130) patients with CVD and diabetes had problem to take proper treatment during COVID-19 infection. Mortality rate was 18% (585 of 3,250) among the participants. About 64% (374 of 585) of the fatalities were found in patients with CVD and COVID-19 followed by 42% (246 of 585) in patients with diabetes and COVID-19. We found that about 96% of the participants had taken 1st dose, 88% 2nd dose and 41% 3rd dose of COVID-19 vaccine (Table 3).

**Table 3:** Distribution of diabetes and cardiovascular disease in different sex and age groups among the study participants.

**Variables**	**Age groups in years (%)**						***N* = 3,250**	***P* value**
	**20–29**	**30–39**	**40–49**	**50–59**	**60–69**	**Above 70**		
**Suffering from cardiovascular diseases (CVD)**								
Yes	48/1,040 (4.6)	216/1,040 (20.8)	222/1,040 (21.3)	265/1,040 (25.5)	143/1,040 (13.8)	135/1,040 (14.0)	1,040/3,250 (32.0)	0.005
No	360/2,210 (16.3)	464/2,210 (21.0)	417/2,210 (18.9)	237/2,210 (10.7)	400/2,210 (18.1)	332/2,210 (15.0)	2,210/3,250 (68.0)	
**Suffering from diabetes**								
Yes	27/780 (3.3)	145/780 (18.6)	142/780 (18.2)	154/780 (19.7)	167/780 (21.4)	145/780 (18.6)	780/3,250 (24.0)	0.005
No	381/2,470 (15.4)	535/2,470 (21.7)	497/2,470 (20.1)	348/2,470 (14.1)	376/2,470 (15.2)	333/2,470 (13.5)	2,470/3,250 (76.0)	
**Suffering from both diabetes and CVD**								
Yes	15/532 (2.8)	103/532 (19.4)	96/532 (18.0)	105/532 (19.7)	123/532 (23.1)	90/532 (16.9)	532/3,250 (16.4)	0.004
No	393/2,718 (14.5)	577/2,718 (21.2)	543/2,718 (20.0)	397/2,718 (14.6)	420/2,718 (15.5)	388/2,718 (14.3)	2,718/3,250 (83.6)	
**Complication related with CVD increased after COVID-19 infection**								
Yes	19/673 (2.8)	105/673 (15.6)	141/673 (21.0)	135/673 (20.1)	131/673 (19.5)	142/673 (21.1)	673/3,250 (20.7)	0.005
No	389/2,577 (15.1)	575/2,577 (22.3)	498/2,577 (19.3)	367/2,577 (14.2)	412/2,577 (16.0)	336/2,577 (13.0)	2,577/3,250 (79.3)	
**Complication related with diabetes increased after COVID-19 infection**								
Yes	5/524 (1.0)	43/524 (8.2)	124/524 (23.7)	78/524 (14.9)	35/524 (6.7)	21/524 (4.0)	429/3,250 (14.4)	0.001
No	405/1,824 (22.2)	468/1,824 (25.7)	304/1,824 (16.7)	215/1,824 (11.8)	237/1,824 (13.0)	195/1,824 (10.7)	1,824/3,250 (85.6)	
**Symptoms of CVD and diabetes worsen after COVID-19 infection**								
Yes	19/320 (5.5)	60/320 (17.3)	81/320 (23.4)	75/320 (21.7)	64/320 (18.5)	47/320 (13.6)	346/3,250 (10.6)	0.005
No	389/2,904 (13.4)	620/2,904 (21.3)	558/2,904 (19.2)	427/2,904 (14.7)	479/2,904 (16.5)	431/2,904 (14.8)	2,904/3,250 (89.4)	
**Co-prevalence of CVD, diabetes and acute long-COVID-19**								
Yes	11/359 (3.1)	57/359 (15.9)	75/359 (20.9)	92/359 (25.6)	68/359 (18.9)	56/359 (15.6)	359/3,250 (11.0)	0.001
No	397/2,891 (13.7)	623/2,891 (21.5)	564/2,891 (19.5)	410/2,891 (14.2)	475/2,891 (16.4)	422/2,891 (14.6)	2,891/3,250 (89.0)	
**Co-prevalence of CVD, diabetes and chronic long-COVID-19**								
Yes	9/387 (2.3)	56/387 (14.5)	79/387 (20.4)	92/387 (23.8)	105/387 (27.1)	46/387 (11.9)	387/3,250 (11.9)	0.005
No	399/2,863 (13.9)	624/2,863 (21.8)	560/2,863 (19.6)	410/2,863 (14.3)	438/2,863 (15.3)	432/2,863 (15.1)	2,863/3,250 (88.1)	
**Fatality in patients with CVD, diabetes and COVID-19**								
Yes	4/235 (1.7)	26/235 (11.1)	43/235 (18.3)	67/235 (28.5)	43/235 (18.3)	52/235 (22.1)	235/3,250 (7.2)	0.005
No	404/3,015 (13.4)	654/3,015 (21.7)	596/3,015 (19.8)	435/3,015 (14.4)	500/3,015 (16.6)	426/3,015 (14.1)	3,015/3,250 (93.3)	
**Taken 1st dose vaccine against COVID-19**								
Yes	360/3,125 (11.5)	656/3,125 (21.0)	619/3,125 (19.8)	491/3,125 (15.7)	527/3,125 (16.9)	472/3,125 (15.1)	3,125/3,250 (96.2)	0.001
No	48/125 (38.4)	24/125 (19.2)	20/125 (16.0)	11/125 (8.8)	16/125 (12.8)	6/125 (4.8)	125/3,250 (3.8)	
**Taken 2nd dose vaccine against COVID-19**								
	322/2,856 (11.3)	603/2,856 (21.1)	581/2,856 (20.3)	431/2,856 (15.1)	507/2,856 (17.8)	412/2,856 (14.4)	2,856/3,250 (87.9)	0.005
	86/394 (21.8)	77/394 (19.5)	58/394 (14.7)	71/394 (18.0)	36/394 (9.1)	66/394 (16.8)	394/3,250 (12.1)	
**Taken 3rd dose vaccine against COVID-19**								
Yes	87/1,347 (6.5)	131/1,347 (9.7)	165/1,347 (12.2)	383/1,347 (28.4)	344/1,347 (25.5)	237/1,347 (17.6)	1,347/3,250 (41.4)	0.002
No	321/1,903 (16.9)	549/1,903 (28.8)	474/1,903 (24.9)	119/1,903 (6.3)	199/1,903 (10.5)	241/1,903 (12.7)	1,903/3,250 (58.6)	
**Treatment problems among patients with CVD and diabetes during COVID-19**								
Yes	11/231 (4.8)	23/231 (10.0)	31/231 (13.4)	36/231 (15.6)	58/231 (25.1)	72/231 (31.2)	231/3,250 (7.1)	0.002
No	397/3,019 (13.2)	657/3,019 (21.8)	608/3,019 (20.1)	466/3,019 (15.4)	485/3,019 (16.1)	406/3,019 (13.4)	3,019/3,250 (92.9)	

*P* < 0.05 was considered statistically significant.

Symptoms were analyzed among patients with acute long-COVID-19, CVD and diabetes (Figure 1C) and chronic long-COVID-19, CVD and diabetes (Figure 1D). Fever (576 of 678) was the most frequent symptoms among patients with acute long-COVID-19 and CVD followed by dry cough (513 of 678), loss of taste or smell (511 of 678) and body aches (345 of 678), respectively (Figure 1C). Similarly, among patients with chronic long-COVID-19, fever (1,695 of 1,707) was the most prevalent followed by dry cough (1,408 of 1,707), loss of taste and smell (1,345 of 1,707) and fatigue (1,234 of 1,707) were most commonly reported (Figure 1D).

### 3.4. Multivariable logistic regression analysis

Multivariable logistic-regression model was used to determine the relationship of different variables with outcome of COVID-19. Independent variables of hospitalization, ICU admission and fatality among patients with COVID-19 and comorbidities and respective odds ratios with 95% confidence intervals were calculated and represented in Table 4. The major predictors of the higher odds of severe health outcome were age >40 years, sex-male, presence of CVD, diabetes, having Charlson Comorbidity Index (CCI) >3, co-prevalence of CVD, diabetes and COVID-19. The highest odds ratio for hospitalization was detected among patients with CCI >3 (OR: 5.53, 95% CI, 5.15–6.14; *p*-value 0.003), followed by positive COVID-19 (OR: 4.63, 95% CI, 3.42–5.37; *p*-value 0.001) and co-existence of COVID-19 and CVD (OR: 4.23, 95% CI, 3.65–5.18; *p*-value 0.002), respectively (Table 4). The risk of ICU admission and fatality was significantly higher among patients with COVID-19 (OR: 4.74, 95% CI, 3.83–5.63; *p*-value 0.001) followed by presence of >3 symptoms (OR: 4.46, 95% CI, 4.02–5.14; *p*-value 0.001), co-prevalence of CVD, diabetes and COVID-19 (OR: 3.65, 95% CI, 2.79–4.24; *p*-value 0.001), and respectively (Table 4). The risk of hospitalization reduced significantly among the vaccinated participants (OR: 0.27, 95% CI, 0.1–0.95; *p*-value 0.001). Notably, vaccinated participants also had lower risk of ICU admission and fatality (OR: 0.38, 95% CI, 0.17–0.74; *p*-value 0.003).

**Table 4:** Multivariable logistic regression analyses to determine the odds of hospitalization and severe outcome among patients with COVID-19 in Bangladesh.

**Variables**	**Odds ratio (95% confidence intervals)**	***P-*value**	**Odds ratio (95% confidence intervals)**	***P-*value**
Age, >60 years vs. < 60 years	4.14 (3.76–5.29)	0.001	3.23 (2.54–4.78)	0.003
Sex, male vs. female	3.34 (2.75–4.58)	0.005	2.64 (1.85–3.43)	0.001
Unemployed vs. employed	1.43 (0.55–2.65)	0.001	1.63 (0.45–2.42)	0.002
CVD and diabetes together vs. CVD alone	3.27 (2.34–4.58)	0.001	2.39 (1.43–3.62)	0.001
CVD and diabetes together vs. diabetes alone	2.43 (1.19–3.53)	0.005	2.14 (1.05–3.79)	0.005
COVID-19 positive vs. COVID-19 negative	4.63 (3.42–5.37)	0.001	4.74 (3.83–5.63)	0.001
CVD and COVID-19 vs. CVD	4.23 (3.65–5.18)	0.002	3.45 (2.63–4.27)	0.001
Diabetes and COVID-19 vs. diabetes	2.26 (1.56–3.14)	0.001	3.15 (2.64–4.02)	0.005
CVD, diabetes and COVID-19 vs. CVD and diabetes	3.19 (2.27–4.69)	0.003	3.65 (2.79–4.24)	0.001
Acute long-COVID-19 vs. chronic long-COVID-19	2.46 (1.76–3.23)	0.001	2.35 (1.54–3.21)	0.007
Acute-long COVID-19, CVD and diabetes vs. CVD and diabetes	3.27 (2.35–4.65)	0.001	3.58 (2.76–4.32)	0.005
Chronic long COVID-19 and CVD vs. CVD	1.56 (0.86–2.41)	0.002	2.31 (1.83–3.23)	0.001
Chronic long COVID-19, CVD and diabetes vs. CVD and diabetes	2.72 (1.75–3.57)	0.003	1.48 (0.9–2.35)	0.048
Vaccinated vs. unvaccinated	0.27 (0.1–0.95)	0.001	0.38 (0.17–0.74)	0.003
Two doses vs. one dose	0.45 (0.13–0.97)	0.002	0.14 (0.09–0.95)	0.001
Three doses vs. two doses	0.56 (0.15–0.99)	0.001	0.68 (0.16–1.21)	0.005
>3 symptoms vs. < 3 symptoms	3.45 (2.63–4.67)	0.003	3.25 (2.74–4.11)	0.005
Worse access to health facilities vs. better access to health facilities	2.76 (2.24–3.53)	0.001	2.53 (2.17–3.26)	0.005
CCI >3 vs. CCI < 3	5.53 (5.15–6.14)	0.005	4.46 (4.02–5.14)	0.001

*P* < 0.05 was considered statistically significant. OR, odds ratio; COPD, chronic obstructive pulmonary disease; CCI, Charlson comorbidity index.

### 3.5. Survival rate analysis

**FIGURE 2:**
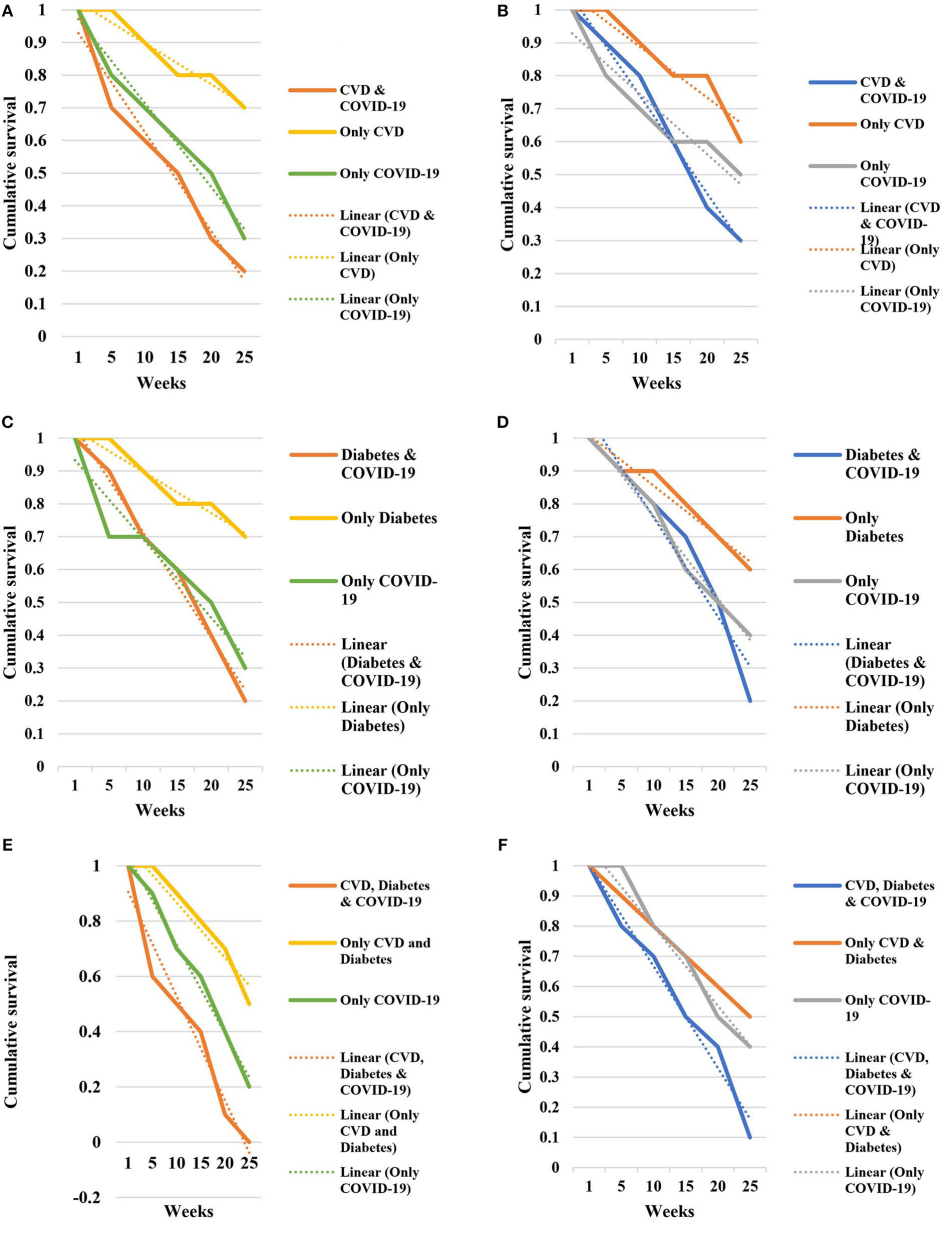
Cumulative survival rate among **(A)** male patients with CVD and COVID-19, **(B)** female patients with CVD and COVID-19, **(C)** male patients with diabetes and COVID-19, **(D)** female patients with diabetes and COVID-19, **(E)** male patients with CVD, diabetes and COVID-19, **(F)** female patients with CVD, diabetes and COVID-19 in Bangladesh.

Cumulative survival analysis of the study population was determined by using the Kaplan-Meier model. The cumulative survival rate of the participants was plotted against the time duration of survival or recovery. The analysis was conducted from week 0 to 25. The Kaplan-Meier model was applied for the data of the patients aged >40 years. We represented the findings for male and female separately. The cumulative survival rate of the male patients without COVID-19 remained above 0.5. Among female patients without COVID-19 the cumulative survival rate was also above 0.5. The cumulative survival rate gradually decreased to 0.0 in 24th week from 1.0 in 1st week among male patients with CVD, diabetes and COVID-19 (Figure 2). The cumulative survival rate among both male and female patients with COVID-19 decreased to 0.2 in the 24th week from 1 in the 1st week. Similarly, the survival rate among female patients with CVD, diabetes and COVID-19 was the lowest 0.1 in the 25th week. Male patients with CVD, diabetes and chronic long-COVID-19 had lower survival rate than female patients at the same time period (Figure 2).

## 4. Discussion

The severity and longtime impact of the COVID-19 pandemic have adversely affected the global health system (8–11). After the onset of the pandemic, it has remained one of the leading causes of global health burden among people. Among many health effects, post-COVID-19 sequelae in the infected is a major problem (11). In this study, we determine the prevalence of patients with acute long-COVID-19 and chronic long-COVID-19 in Bangladesh, and impact of COVID-19 on pre-existing cardiovascular disease and diabetes among them. We specified the predictors associated with poor health outcome, hospitalization, ICU admission and fatality among patients with COVID-19. We found that 73.4% of the participants were positive for COVID-19. About 28.4% of the patients were suffering from acute long-COVID-19 and 71.6% from chronic long-COVID-19 in Bangladesh. The prevalence of long COVID-19 reported in this study is higher than any of the previous studies (12–20). The probable reason might be the study cohort had pre-existing comorbidities, high prevalence of COVID-19, circulation of omicron variant (90%) during the study, and higher population density in the study regions. Nearly 29% of the patients had symptoms of COVID-19 for 13–24 weeks, 25.6% for 25–48 weeks and 17% for >48 weeks. These findings are one of the first reports of long COVID-19 in Bangladesh. The prevalence of COVID-19 is also high in this study compared to the previous studies (8–12, 16, 18–20).

In the comorbidity analysis, we found that cardiovascular (CVD) disease was the most prevalent (32%) followed by diabetes (24%; Type 2 diabetes mellitus was 63.7% and Type 1 was 36.3%). Previous studies have reported the prevalence of diabetes between 10% and 100% among patients with COVID-19 (12–19, 21–24). Reported median glycaemia was 9.3 mmol/L (IQR 7·2–10·7) among the patients with COVID-19, which is in good agreement with previous studies (12, 16, 19). Previous studies have reported the prevalence of cardiovascular disease between 2% and 40% in patients with COVID-19 in different countries (16, 19, 21–25). Coronary artery disease was the most frequent cardiovascular disease followed by hypertension, cardiac arrhythmia and congestive heart failure, respectively. These findings are in good agreement with previous studies in Bangladesh, Saudi Arabia and China (11–19, 21–27). Co-prevalence of CVD, diabetes and COVID-19 was involved in majority of fatality (95%) reported among the participants, which is in good agreement with previous studies in Bangladesh, China and other countries (16–19, 22–28). We detected 16.4% patients of COVID-19 had both CVD and diabetes. Health complications associated with CVD increased among 64.7% patients and diabetes among 55% patients after getting COVID-19 infection. These findings are supported by the previous studies in different countries (16, 18, 19, 22–31). Co-prevalence of CVD, diabetes and acute long-COVID-19 was found among 11% and co-prevalence of CVD, diabetes and chronic long-COVID-19 were detected among 11.9% patients. These findings will add knowledge on long-COVID-19 and comorbidity in Saudi Arabia for the first time. This study reflected previous studies focusing single and combined impacts of comorbidities like CVD, diabetes, COPD and demographic factors like age and sex on the outcome including hospitalization, ICU admission and fatality (11, 13–19, 22, 24–32).

Symptoms associated with acute and chronic long-COVID-19 were analyzed. Among the patients with acute long-COVID-19, fever (97%) was most prevalent followed by sore throat (89%), loss of taste or smell (86%) and dry cough (79%), respectively. Further, fever (95%) was the most prevalent symptoms followed by dry cough (88%), loss of taste and smell (83%) and fatigue (78%) among the patients with chronic long-COVID-19. Reappearance of different of symptoms of COVID-19 after recovery from infection had prolonged health impact on the patients with CVD and diabetes (8–12). However, the distribution of prevalence of different symptoms were similar among the COVID-19 patients and those with long-COVID-19. These findings will add new knowledge on the clinical spectrum of COVID-19 in Bangladesh.

We detected the highest odds ratio for hospitalization among patients with CCI >3 (OR: 5.53, 95% CI, 5.15–6.14) and COVID-19 (OR: 4.63, 95% CI, 3.42–5.37). Patients with COVID-19 (OR: 4.74, 95% CI, 3.38–5.63) and co-prevalence of CVD, diabetes and COVID-19 (OR: 3.65, 95% CI, 2.79–4.24) had higher risk of ICU admission and fatality. We also found that vaccinated people had lower risk of hospitalization (OR: 0.27, 95% CI, 0.1–0.95; *p*-value 0.001), ICU admission and fatality (OR: 0.38, 95% CI, 0.17–0.74; *p*-value 0.003). These findings are in good similarity with previous studies (11–19, 22–28, 31–33). In a similar study in Bangladesh, the authors also reported higher odds of fatality and ICU admission among patients with CVD, diabetes and COVID-19 (16, 19). We found higher risk of fatality in male patients aged above 40 years suffering from CVD, diabetes and COVID-19 (OR: 3.41, 95% CI, 2.62–4.83). These findings are in similarity with previous reports worldwide (11–19, 24–33). Presence of acute and chronic long-COVID-19 also increased the risk of hospitalizations, ICU admission and fatality among the participants. In similar with previous studies, we also found that female and participants aged below 30 years had lower risk of developing long-COVID-19 associated severity (16, 19). These might be due to their stronger immunity against viral infection, which needs detail analysis in future (16, 19).

We found that presence of COVID-19 contributed to the development of serious health outcome among patients with CVD and diabetes in Bangladesh. Majority of the fatality were associated with COVID-19, CVD and diabetes among the patients. However, we found lower fatality rate among the patients with chronic long-COVID-19 compared with patients with acute long-COVID-19. These findings are relatively new for data of Bangladesh. Presence of symptoms of COVID-19 for longer period and reappearance after certain time might affect the pre-existing CVD and diabetes. Studies have found that infection of COVID-19 have contributed to development of diabetes for certain period among the patients (16–19, 21–28). Further, studies have also reported that infection with COVID-19 might worsen the existing cardiovascular disease in patients. Certain medicines used to treat cardiovascular disease might have roles in poor health outcome among the COVID-19 patients (16, 19, 24–29, 31–33). Presence of previous CVD and diabetes increased the risk of poor health outcome and fatality rate after COVID-19 infection. Inversely, infection of COVID-19 has also triggered different adverse health impact in patients with CVD and diabetes. Previous studies have confirmed that COVID-19 infection has increased incidence of cardiac arrest, cardiomyopathy, myocardial infarction, and cardiac arrhythmias (13–19, 23, 26–33).

The main limitation of the study was the limited size of the population. Further, we could not include data on real-time hemoglobin A1c (HbA1c) and CVD disease conditions of the patients. Data on the clinical manifestations were missing for some of the patients and some of the data were self-reported.

This is one of the first report of long-COVID-19 and associated health outcome in Bangladesh. In this study we reported high prevalence of chronic long-COVID-19 among patients in Bangladesh. Co-existence of CVD, diabetes and COVID-19 have significantly contributed to hospitalization, ICU admission and fatality among the participants. This study will add knowledge in understanding the long-time impact of COVID-19 and health burden of preexisting CVD and diabetes. Further, these findings will provide baseline data for future studies to reveal the exact mechanism of complicated health outcome and impact of COVID-19 among patients with CVD and diabetes.

## Data availability statement

The raw data supporting the conclusions of this article will be made available by the authors, without undue reservation.

## Ethics statement

The studies involving humans were approved by the Biosafety, Biosecurity, and Ethical Committee (BBEC) of Jahangirnagar University, Savar, Dhaka-1342, Bangladesh. The protocol number approved by the Ethics Committee is BBEC, JU/M 2021/COVID-19/(2)1. The studies were conducted in accordance with the local legislation and institutional requirements. The participants provided their written informed consent to participate in this study.

## Author contributions

NadS: conceptualization (lead), data curation (lead), formal analysis (lead), investigation (equal), methodology (lead), project administration (lead), software (lead), validation (lead), writing—original draft (lead), and writing—review and editing (lead). NazS: data curation (equal), methodology (equal), investigation (equal), methodology (lead), project administration (lead), software (lead), validation (lead), writing—original draft (lead), and writing—review and editing (lead). AK: validation (equal), writing—original draft (supporting), data curation (equal), and investigation (equal). IH: writing—review and editing (equal) and formal analysis (supporting). FA: software (lead) and writing—review and editing (equal). KA: writing—original draft preparation (supporting) and methodology (supporting). ID: data curation (equal), project administration (equal), software (equal), and validation (equal). DV: data curation (equal), software (equal), and validation (equal). AC: data curation (equal), project administration (equal), software (equal), and validation (equal). AP: software (lead) and validation (lead). SD: project administration (lead), software (lead), validation (lead), and writing—review and editing (lead). All authors contributed to the article and approved the submitted version.
