# Supplementary material for: Prevalence and impact of long COVID-19 among patients with diabetes and cardiovascular diseases in Bangladesh
Source: Front Public Health. 2023 Oct 27;11:1222868. doi: 10.3389/fpubh.2023.1222868 (PMC10641795; doi:10.3389/fpubh.2023.1222868)
Supplement: Supplementary file 1 [file Table_1.DOCX]

**Table a.** Occupation of study participants.

| **Occupation** | **Male** | **Female** | **Total** |
| --- | --- | --- | --- |
| Physician | 203/303 (67) | 100/303 (33) | 303/3250 (9.3) |
| Teacher | 225/325 (69.2) | 100/325 (30.8) | 325/3250 (10) |
| Researcher | 213/312 (68.3) | 99/312 (31.7) | 312/3250 (9.6) |
| Farmer | 145/217 (66.8) | 72/217 (33.2) | 217/3250 (6.7) |
| Nurse | 154/231 (66.7) | 77/231 (33.3) | 231/3250 (7.1) |
| Student | 388/537 (72.3) | 149/537 (27.7) | 537/3250 (16.5) |
| Journalist | 74/85 (87.1) | 11/85 (12.9) | 85/3250 (2.6) |
| Political person | 82/97 (84.5) | 15/97 (15.5) | 97/3250 (3) |
| Lawyer | 102/151 (67.5) | 49/151 (32.5) | 151/3250 (4.6) |
| Police | 85/112 (75.9) | 27/112 (24.1) | 112/3250 (3.4) |
| Banker | 93/121 (76.9) | 28/121 (23.1) | 121/3250 (3.7) |
| Administrative  Officer | 105/136 (77.2) | 31/136 (22.8) | 136/3250 (4.2) |
| Private employee | 213/302 (70.5) | 89/302 (29.5) | 302/3250 (9.3) |
| Driver | 89/114 (78.1) | 25/114 (21.9) | 114/3250 (3.5) |
| Businessman | 80/105 (76.2) | 25/105 (23.8) | 105/3250 (3.2) |
| Others | 89/102 (87.3) | 13/102 (12.7) | 102/3250 (3.1) |
